# Phosphorus Solubilization by *Bacillus* Species

**DOI:** 10.3390/molecules23112897

**Published:** 2018-11-06

**Authors:** Agnieszka Saeid, Ewelina Prochownik, Justyna Dobrowolska-Iwanek

**Affiliations:** 1 Department of Advanced Material Technologies, Faculty of Chemistry, Wroclaw University of Science and Technology, Smoluchowskiego 25, 50-372 Wroclaw, Poland; 2 Department of Food Chemistry and Nutrition, Jagiellonian University Medical College, 9 Medyczna Street, 30-688 Krakow, Poland; ewelinakica@op.pl (E.P.); justyna.dobrowolska-iwanek@uj.edu.pl (J.D.-I.)

**Keywords:** microbial solubilization, *Bacillus megaterium*, *Bacillus subtilis*, *Bacillus cereus*, organic acids, solubilization factor

## Abstract

Microbial solubilization applies the natural ability of a microorganism to liberate phosphorus from unavailable structures. The main mechanism recognized to be responsible for the solubilization of phosphorus is the production of different types of organic acids. Three kinds of *Bacillus* species and three types of raw materials (poultry bones, fish bones, and ash) were tested for solubilization. The following parameters were compared for all discussed cases: pH, specific growth rate, solubilization factor, released phosphorus concentration, and total and individual concentration of organic acids. Utilization of ash brought about the highest specific and maximum specific growth rates. A decrease in pH was observed in most of the discussed cases with the exception of fish bones. At the same time, fish bones had the highest concentration of released P_2_O_5_ and the highest total concentration of produced organic acids (gluconic, lactic, acetic, succinic, and propionic) in all discussed cases. The tested *Bacillus* species produced the mentioned acids with the exception of *B. megaterium*, where propionic acid was not present. The lactic and acetic acids were those produced in the highest amount. The kind of raw materials and type of *Bacillus* species used in solubilization had a strong influence on the kind of organic acids that were detected in the broth culture and its total concentration, which had a direct influence on the amount of released phosphorus. The combination of *Bacillus megaterium* with the fish bones at 5 g/L is proposed as the pair that gives the highest concentration of released phosphorus (483 ± 5 mg/L).

## 1. Introduction

The main raw materials having industrial importance in phosphorus fertilizer production are phosphorite and apatite. Mineral raw materials are non-renewable sources of phosphorus, and their resources are limited and gradually depleted. In May 2014, the European Commission published an updated list of raw materials that are crucial for the global economy. Among the six new materials attached to the 2011 list, phosphorus ores were mentioned [1]. In December 2015, the European Commission adopted a package for the economy in the closed cycle, which covers the entire product lifecycle, waste management, and recyclable materials. One of the activities toward the implementation of such a model is a proposal to amend the regulation on fertilizers, especially those that are organic and derived from waste because, so far, these products cannot compete with conventional fertilizers produced from raw materials. Phosphorus flow analysis shows that the largest loss of this element occurs in waste streams [2]. The development of methods for the recovery of phosphorus from waste is, therefore, a key measure aimed at improving the balance of phosphorus, both globally and locally. Promising alternatives of phosphorus sources are by-products of wastewater treatment, such as sewage sludge and ashes, as well as industrial side-streams [3]. 

Alternative phosphorus source utilization requires chemical processing since the phosphorus present in those materials is not available to plants and cannot be directly applied to the soil. A possible alternative for treating phosphorus-bearing waste flux by acids obtained from the chemical industry [4] is the utilization of organic acids originating in microorganisms present in the soil. One of the most dominant rhizospheric bacteria *Bacillus* (*B. subtilis*, *B. cereus*, *B. thuringiensis*, *B. pumilus*, *B. megaterium*, etc.) developed different mechanisms to enhance plant growth by increasing availability of the nutrient [5]. One of the main mechanisms recognized as responsible for releasing available forms of phosphorus to plants in the soil is the production of organic acids (solubilization of insoluble inorganic phosphate compounds such as tricalcium phosphate, dicalcium phosphate, hydroxyapatite, and rock phosphate) [6,7] and the increase in activity of acid phosphatases (mineralization of organic phosphorous) [8]. 

The strategy to solubilize the unavailable form of phosphorus (PO_4_^3−^) from soil to available (HPO_4_^2−^ and H_2_PO_4_^−^) [9] is also used by plants themselves [10,11]. Plants boost the efficiency of phosphorus acquisition by enhancing the activity of acid phosphatase, an enzyme that is responsible for phosphorus solubilization, thus significantly increasing the quantity of root acid exudation. The ability of the root to recognize and specifically respond to the nutrient status of soil through an increased production of organic acids was previously described [12] and proven [13]. Phosphate-solubilizing microorganisms (PSM) are very important to the plants under phosphorus stress [14]. In certain cases, phosphate solubilization is induced by phosphate starvation [14].

Organic acid concentration decreases with increasing P supply. Organic acids are deemed as major compounds that are exudates by the root system [10] and, in this way, can displace phosphate from soil particles that are not available to plants, thus increasing phosphate availability [11,12].

Some of the most promising waste streams that could potentially deliver phosphorus are ashes originating from sludge incineration in wastewater treatment plants with the third stage of biological treatment, and bones that are characterized by a high phosphorus content [15]. Biotechnological valorization of these kinds of secondary raw materials, which are very often recognized as wastes difficult to utilize, is possible through the application of the natural ability of soil microbiota to transform phosphorus from non-available into available, which was previously described in the literature [16,17,18,19,20,21]. Worldwide, soils are supplemented with inorganic P as chemical fertilizers to support crop production; however, repeated use of fertilizers deteriorates soil quality. Therefore, the present tendency is to shift toward a more sustainable agriculture [22]. A biological approach for liberating phosphate from P-bearing materials through organic acid produced by soil microorganisms was proposed as a less expensive and lower-energy technique compared with its conventional chemical counterpart [21,22].

The production of organic acids, which are recognized as a major mechanism responsible for releasing phosphorus from hydroxyapatite structures, and their identification are crucial when new kinds of raw materials are proposed for valorization. In this paper, three renewable resources of phosphorus and one non-renewable were used as a source of phosphorus for solubilization performed by three different kinds of *Bacillus* species. Our objectives were to identify and determine the concentration of the organic acid composition of the filtrates and to test if the organic acids were capable of solubilizing phosphate delivered in the form of a low-quality phosphorus resource. The influence on bacterial cell growth, changes in pH, and concentration of released P_2_O_5_ were evaluated and discussed in the aspect of produced acids, in the view of their possible further utilization in the production of phosphorus fertilizers.

## 2. Results

### 2.1. Growth of Bacillus

The phosphorus in the consortium trial with no addition of secondary raw materials was delivered in the form of Ca_3_(PO_4_)_2_; in the rest of trials, where all considered P-bearing materials were used, phosphorus was replaced. At the same time, the goal of this part of the experiments was to evaluate the eventual toxic influence of used P-bearing materials on the growth of bacteria. Microorganism growth was monitored for four days, which is the time, based on previous experiments [15,23], necessary to reach the *Bacillus* cell concentration plateau (stationary growth). Table S1 (Supplementary Materials) shows measured values of specific growth rates. In most cases, the increase in doses of raw materials, both renewable (poultry bones, fish bones, and ash) and non-renewable (e.g., phosphate rock), resulted in the increase in the specific growth rate *μ* (1/day). Only in the case of phosphate rock, when solubilization was performed by *B. cereus* and *B. subtilis*, was the inhibition observed as a result of an increase in applied doses in the growth medium. When the consortium was compared with the standard medium as a control, a value of 0.130 1/h was observed, closely similar to the consortium with fish bones (0.132 1/h), probably due to the fish bones delivering the phosphorus in a form more available to bacteria when compared with the rest of the considered materials. When taking into account the influence of the source of phosphorus on the growth of bacteria strains within the consortium culture, it is not possible to conclude which strain played the role of dominant if any, as the measure of growth was based only on the optical density (OD) parameter. Nevertheless, it can be stated that fish bones serve as a better source of nutrients since *μ* was the highest (Table S1, Supplementary Materials). Similar to results obtained in monoculture broths, in the consortium, a significantly lower *μ* was found when phosphate rock was used.

When considering the monocultures and taking into account the efficient utilization of secondary raw materials as a source of nutrients necessary for bacterial cell growth, ash seems to be the best choice when compared to poultry or fish bones and phosphate rock. The highest specific growth rate *μ*, 1/day was found when the ash was used as a source of phosphorus in all studied bacterial strains (Table S1, Supplementary Materials, bolded values). Interesting is that the similar results in specific growth rate were found in the monocultures when ash was used as a source of phosphorus: *B. subtilis* with ash (0.134 1/day), *B. cereus* with ash (0.143 1/day), and *B. megaterium* with ash (0.139 1/day).

When the influence of the dose of the applied raw material on specific growth rate was taken into account, the experimental data were described by two different models: Monod (Equation (1)) and Haldane (Equation (2)). Haldane was used when the inhibition was observed. All evaluated model parameters are listed in Table 1.

(1) $$\mu =\mu_{max}\frac{S}{K_{S}+S^{\prime }}$$

where *μ_max_* is the maximal specific growth rate (1/day), *S* is the concentration of substrate (mg/L), and *K_S_* is the Monod constant (mg/L); this constant expresses the concentration of substrate that results in the specific growth rate equal to ½ *μ_max_* day^−1^. The Haldane model was used to describe the growth of microorganisms under toxic conditions as a result of too high concentrations of phosphate raw materials.

(2) $$\mu =\mu_{max}\frac{S}{K_{S}+S+\frac{S^{2\prime }}{K_{I}}}$$

where *K_S_* is the inhibition constant, which expresses the degree of inhibition (mg/L).

Haldane’s model parameters were evaluated for two cases (*B. cereus* and *B. subtilis*), where phosphate rock (PR) was applied as a source of phosphorus because, only in those cases, was the inhibition in the specific growth rate observed. The inhibition of growth of mentioned *Bacillus* species was probably due to the origin and composition of phosphate rock. The presence of toxic substances such as heavy metals [24] could be the reason for lowering the specific growth rate when the higher dose was applied. In other cases, the inhibition was not observed and experimental data were described by the Monod model parameters. Similar to previous findings, the highest maximal specific growth rate was also found for ash. This observation agrees with other findings described in the literature, where it was reported that ashes could enhance *Bacillus subtilis* growth [25].

A decrease in pH was observed in most studied cases as a result of the organic acid production (Table S1, Supplementary Materials, last column) [16,17,18,19,20,21]. On the other hand, a pH increase was observed when fish bones were used (Table S1, Supplementary Materials, last column). Similar findings were observed in the case of solubilization performed by a consortium of *Bacillus* species. In the case of fish bones, an increase in pH was also found. This finding is not in line with the previous findings, which showed a pH decrease when fish bones were used [15]. The highest pH changes were observed when the raw material dose was the smallest, probably because, when the raw material dose was the highest, the compound concentrations that could possibly neutralize the produced acids were also higher, thus limiting the pH changes. 

The real effectiveness of the solubilization was expressed as the solubilization factor (SF, %) defined as the ratio (expressed as a percentage) of soluble P_2_O_5_ present in the solution to phosphorus (expressed as P_2_O_5_) introduced to the solubilization medium in solid form (12.8%—poultry bones; 19.6%—fish bones; 13.4—ash; 34.2%—phosphate rock). Table S2 (Supplementary Materials) presents the correlation matrix that expresses the relationship between pH, P_2_O_5_, and solubilization factor (SF). The presented data confirm a strong correlation between pH and solubilization efficiency, expressed here as P_2_O_5_ concentrations in the solution during solubilization and SF. 

### 2.2. Concentration of Phosphorus (Expressed as P_2_O_5_)

The production of organic acids results in acidification of the microbial cell and its surroundings by decreasing pH. The amount of soluble phosphate released depends on the strength and type of produced acids [14]. To describe the changes in P_2_O_5_ concentrations during solubilization, a previously proposed model was used [15]:

(3) $$C_{P_{2}O_{5}}=f\left(t\right)=\frac{C_{P_{2}O_{5}}^{max}}{1+b\cdot exp^{-k\cdot t}},$$

where $C_{P_{2}O_{5}}^{max}$ (mg/L) is the maximum P_2_O_5_ concentration, *b* is a constant that expresses the time when $C_{P_{2}O_{5}}$ is equal to ½ of $C_{P_{2}O_{5}}^{max}$, and *k* (1/day) is a constant for the variable slope, which is called the Hill slope. When *k* is greater, the curve changes more rapidly, i.e., solubilization is faster. 

Table 2 displays the measured values of all parameters of the proposed model, as well as the *p*-value and errors that express the fit of the model to the experimental data. When poultry bones were used as a phosphorus source, the highest $C_{P_{2}O_{5}}^{max}$ (279 mg/L) was found when solubilization was performed by *B. megaterium* and when the bone dose was 30 g/L. The smallest $C_{P_{2}O_{5}}^{max}$ (42.6 mg/L) was evaluated for *B. cereus* with the bone dose equal to 30 g/L. Fish bones used as a renewable source of phosphorus resulted in the highest measured value of model parameter $C_{P_{2}O_{5}}^{max}$. For example, when *B. megaterium* carried out solubilization of fish bones used at 5 g/L, the $C_{P_{2}O_{5}}^{max}$ was equal to 483 mg/L. At the same time, the smallest evaluated value of $C_{P_{2}O_{5}}^{max}$ (213 mg/L) for fish bones was found also for *B. megaterium*. Utilization of ash and phosphate rock in solubilization resulted in the comparable lowest values of the evaluated model parameter $C_{P_{2}O_{5}}^{max}$. The highest for ash was 85.4 mg/L, while that for phosphate rock was 41.2 mg/L. On the other hand, the lowest value of $C_{P_{2}O_{5}}^{max}$ for ash used in the solubilization process was 27.3 mg/L, and that for phosphate rock was 29.5 mg/L. Similarly, in the case of the consortium, the highest $C_{P_{2}O_{5}}^{max}$ was obtained when fish bones were used (414 ± 9 mg/L) and this result was comparable with the efficiency of the solubilization process performed by *B. megaterium* on the fish bones (483 ± 5 mg/L).

Summarizing the possibilities of four different sources of phosphorus in solubilization as a valorization method of secondary wastes into phosphorus fertilizers, fish bones seem to be one that ensures the highest possible concentrations of P_2_O_5_. 

At the same time, the highest specific growth rate *μ* (1/day) and maximal specific growth rate were found when ash was used as a phosphorus source in all bacterial strains under study. This interesting finding can be explained by the fact that the compounds delivered by the ash resulted in the growth but not the synthesis of organic acids, which is used for increasing the availability of phosphorus in the natural environment of soil. The forms of phosphorus in the biological origin of raw materials such as poultry bones and fish bones differ significantly from the forms delivered in the ash. The hydroxyapatite from bones is susceptible to organic acids. The production of acids was probably limited because of the lack of organic compounds in the ash; however, at the same time, all compounds necessary for growth were delivered.

### 2.3. Organic Acids

**Figure 1:**
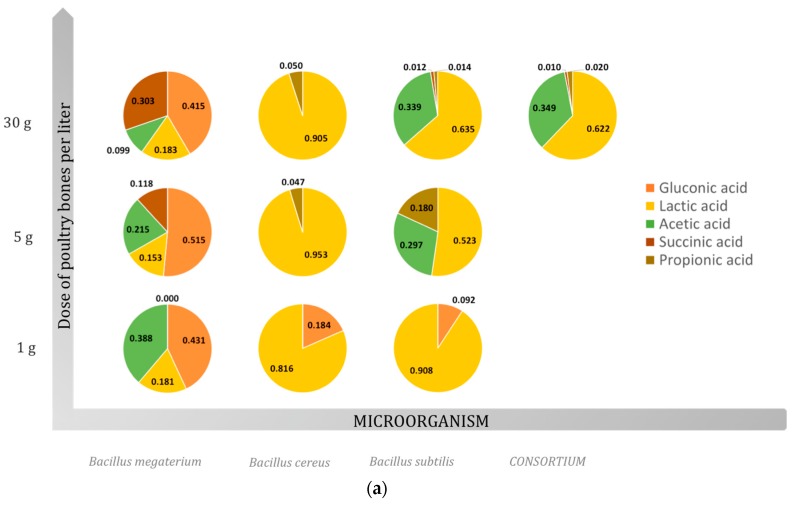

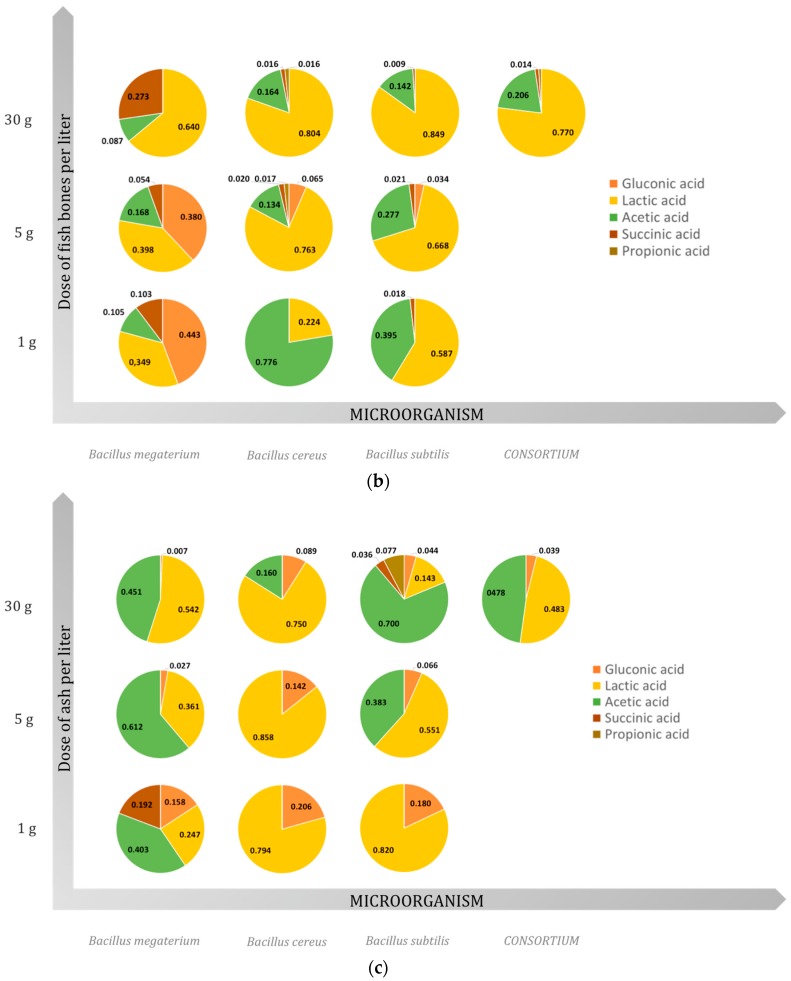

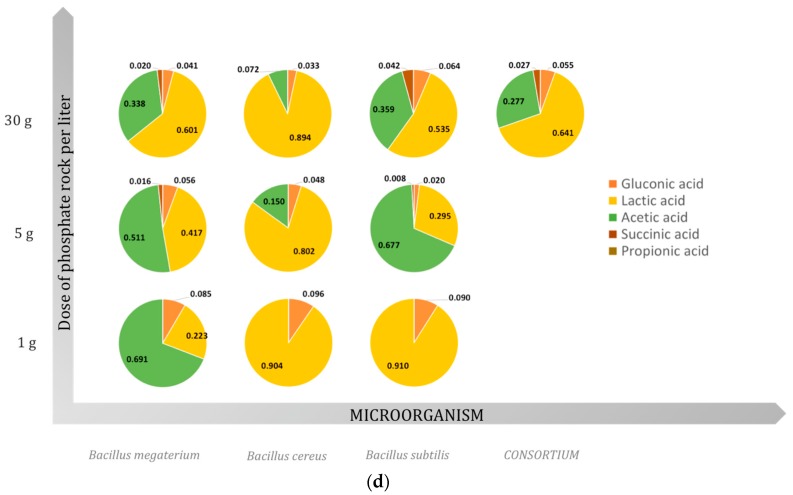
Contribution (mass fraction) of individual acids in relation to the obtained spectrum of organic acids (mg/L) in a microbial broth of *Bacillus* species in solubilization performed with the utilization of four sources of phosphorus (*N* = 3): (**a**) poultry bones; (**b**) fish bones; (**c**) ash; (**d**) phosphate rock.

The main mechanism of phosphate solubilization is the production of organic acids. Gluconic, formic, 2-ketogluconic, citric, oxalic, lactic, isovaleric, succinic, glycolic, and acetic acids are among some produced by phosphate-solubilizing microorganisms [26]. In addition to those, pyruvic, malic, fumaric, and alpha-ketoglutaric acids were also identified [27,28]. The carbon source is crucial for the nature and type of acid that is produced by phosphate-solubilizing bacteria [14]. The production of organic acids results in the lowering of pH in the surroundings. The lower pH of the medium suggests the release of organic acids by the P-solubilizing microorganisms via the direct oxidation pathway that occurs on the outer face of the cytoplasmic membrane [26]. In the case of the performed experiments, gluconic, lactic, acetic, succinic, and propionic acids were detected the in culture broth. A strong influence of the material added to the culture broth on the spectrum of produced acids was found. Figure 1 presents the fractional share of individual acids in the culture broth, while Table 3 shows the concentrations of specific organic acids detected in the studied cases. 

#### 2.3.1. Gluconic Acid

Gluconic acid was produced by all tested *Bacillus* species with a variation of detected concentrations dependent on the type of raw material used and tested doses. Its presence in broth medium was also reported with citric acids by Reyes et al. [29] in the case of *Penicillium rugulosum*, and by Oteino et al. [6] in the case of *Pseudomonas fluorescens*.

The role of gluconic acid in the liberation of phosphates from tricalcium phosphate (TCP) is not only acidification as mentioned before but also chelation, that enables it to form insoluble complexes with Ca^2+^ [30].

In the case of *B. megaterium* with the poultry bones, an increase in gluconic acid concentrations co-occurred with an increase in bone doses (*r* = 0.940). A similar relationship was found for *B. megaterium* when phosphate rock was applied. In a few cases, it was recognized that higher doses of raw material inhibited the production of gluconic acid. The highest concentrations of gluconic acid were found for *B. megaterium* at 30 g/L poultry bones (11.8 ± 0.1 mmol/L) and for fish bones at 5 g/L, while Mardad et al. [27] reported significantly higher concentrations of gluconic acid extending to 55.4 mM.

#### 2.3.2. Lactic Acid

Lactic acid was produced by all tested *Bacillus* species and for all tested doses of renewable phosphorus raw materials. What is more, the inhibition effect of increased doses of raw material that was observed for the cases mentioned above for gluconic acid was not observed in the case of lactic acid. For *B. megaterium* and *B. cereus*, high correlation coefficients (above 0.950) were found between the doses of raw materials and the detected concentrations of acids in the broth medium. Only in the case of *B. subtilis* when ash and phosphate rock was used as a source of phosphorus was the relationship not so obvious, probably due to the inorganic form of raw materials. The highest concentration of lactic acid (36.7 ± 0.4 mmol/L) was found for *B. subtilis* when fish bones were applied as a phosphorus raw material. A similar result was obtained for the consortium when fish bones were used (37.7 ± 0.1 mmol/L). 

It seems that the fish bones deliver nutrient compounds that stimulate the production of lactic acid, as the concentration of lactic acid in all treatments with fish bones were similar also highest when compared with other sources of phosphorus. According to Table 3, treatments with fish bones applied at the highest dose (30 g/L) resulted in the following lactic acid concentrations: 27.5 mmol/L (*B. megaterium*), 35.5 mmol/L (*B. cereus*), 36.7 mmol/L (*B. subtilis*), and 37.7 mmol/L (consortium).

#### 2.3.3. Acetic Acid

Acetic acid was produced by all studied bacterial strains, with a variation in detected concentration that was strongly correlated with the kind of *Bacillus* strain and the raw materials used in the experiments. When the solubilization was performed by consortium, the highest concentration was found when poultry bones were used (12.9 ± 0.3 mmol/L); on the other hand, when pure cultures of specific *Bacillus* were studied, the highest concentration of acetic acid was detected for *Bacillus cereus* when fish bones were used at the smallest dose 1 g/L (15.2 ± 0.3 mmol/L). In the case of *Bacillus cereus*, acetic acid was not produced when poultry bones were used as a source of phosphorus. What is more, it was produced by *B. cereus* when higher doses of inorganic sources of phosphorus were used (ash and phosphate rock).

#### 2.3.4. Succinic Acid

Succinic acid was not produced in all studied cases. Only in the case of *B. megaterium* and *B. subtilis* was this acid detected, and an increase in the concentrations of produced acid co-occurring with an increase in dose was observed. In the case of *B. cereus*, succinic acid was found only in the case where fish bones were used. The highest concentration of succinic acid (11.8 ± 0.018 mmol/L) was found for the culture of *B. megaterium* with fish bones in the growth medium. In previously conducted experiments, when the solubilization process was performed by *B. megaterium* with the utilization of poultry bones, succinic acids were not detected [15].

#### 2.3.5. Propionic Acid

The amount of propionic acid produced depended on the microorganism, which was in line with previous findings [14]. Propionic acid was not detected in the *B. megaterium* culture under any tested conditions. The addition of higher levels of poultry bones to the growth medium of *B. subtilis* and *B. cereus* produced propionic acid. Higher doses of raw material used in the growth medium resulted in higher concentrations of propionic acid being detected. The correlation coefficient between dose and acid concentration in both cases was higher than 0.90. In the case of *B. subtilis*, propionic acid was found when poultry bones or ash were used. Detected levels of propionic acid were much lower when compared with other acids that were found in the culture broth. The highest concentrations of propionic acid (2.67 ± 0.05 mmol/L) were produced by *B. subtilis* when 5 g/L poultry bones were used in the solubilization. 

### 2.4. Total Concentrations of Produced Organic Acids vs. Concentrations of P_2_O_5_

Microbial metabolites such as organic acids convert phosphate into soluble forms, by chelating the cations bound to phosphate through their hydroxyl and carboxyl groups [27]. The literature reports that solubilizing rock phosphate with phosphate-solubilizing bacteria (PSB) is influenced by higher concentrations of organic acids required to mobilize major quantities of insoluble phosphate into the solution via the direct oxidation of phosphates, which occurs on the outer face of the cytoplasmic membrane. Organic acids, pH, and bacterial community affected both solubilization and P availability [31]. The variance in the presence of P_2_O_5_ in the solutions in the described experiments could be explained by the variation in synthesized organic acids, which is directly affected by the kind of bacterial strain, as well as by the kind of raw material used as a source of phosphorus in the solubilization. A statistical evaluation of experimental data pointed at the statistically significant correlation between $C_{organic\;acids}^{total}$ and $C_{P_{2}O_{5}}$, where higher total concentrations of organic acids produced in the solubilization process resulted in higher soluble P_2_O_5_ being found in the solution (*N* = 36, *r* = 0.64, *p* = 0.000026) (Table 4). The combination of *Bacillus megaterium* with the fish bones at 5 g/L was the pair that gave the highest concentration of released phosphorus (expressed as P_2_O_5_; 483 ± 5 mg/L) (Table 2).

The total organic acid concentrations produced by tested PSB ranged from 2.3 mmol/L (*B. subtilis*, ash at 1 g/L) to 48.9 mmol/L (consortium, fish bones at 30 g/L). Performing the solubilization process with the consortium of *Bacillus* species resulted in the highest total organic acid concentration (48.9 mmol/L). In the case of poultry bones, as mentioned before, the concentration was the highest when compared with all discussed cases (37.2 mmol/L), while it was significantly lower for ash (9.6 mmol/L) and phosphate rock (13.7 mmol/L).

Hayat et al. [32] reported concentrations of organic acids in their solubilization experiments a few times lower (10.2 mM). Wei et al. [33] reported that the total concentrations of organic acids reached the maximum at the cooling stage in all treatments during composting, ranging from 45.7 mg/g to 82.7 mg/g, also a few times smaller than the results reported herein. 

**Figure 2:**
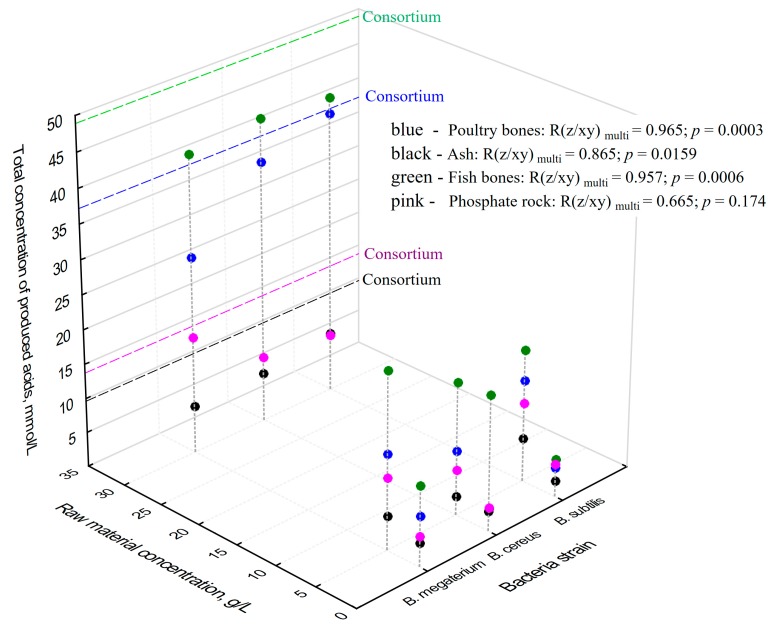
The influence of dose of raw material (g/L) and kind of bacterial strain on the total amount of produced acids (mmol/L): poultry bones (blue), ash (black), fish bones (green), phosphate rock (pink). Dashed lines represent the concentrations of produced acids obtained in the culture of microbial consortium when 30 g/L of raw material was used.

When the total of detected concentrations of produced organic acids (expressed as mmol/L) is taken into account and compared with the applied dose of raw material and with the kind of species of *Bacillus*, a very strong relationship between them can be found (Figure 2), $C_{organic\;acids}^{total}=f\left(dose\right)$. In the case of poultry bones (blue circles) for all tested microorganisms, an increase in bone dose resulted in a higher amount of produced organic acids. The direction factor obtained for the regression curve ($C_{organic\;acids}^{total}=f\left(dose\right)$) was highest for *B. cereus* (1.18, *R*^2^ = 0.999) and for *B. subtilis* (1.18, *R*^2^ = 0.976), while, for *B. megaterium*, it was two times lower (0.679, *R*^2^ = 0.968), which can be interpreted as a lower adaptation to higher concentrations of phosphorus resources in the growth medium. 

Another organic resource used in the experiments was fish bones. Similar relationships, when compared to poultry bones findings, were recognized, although higher concentrations of acids were detected (44 mmol/L). The following direction factors ($C_{organic\;acids}^{total}=f\left(dose\right)$) were evaluated: *B. cereus* 0.899 (*R*^2^ = 0.979); *B. subtilis* 1.18 (*R*^2^ = 0.944); *B. megaterium* 0.948 (*R*^2^ = 0.895) (Figure 2).

In the case of ash used as a source of phosphorus in solubilization, the total concentration of produced acids was 2–4 times smaller when compared with biological resources (poultry or fish bones). At the same time, an increase in the amount of produced acid was found with an increase in the applied dose of phosphorus raw material, just as in the case of bones. Similar to poultry bones, the highest direction factor of the regression curve, $C_{organic\;acids}^{total}=f\left(dose\right)$, was found for *B. cereus* (0.153, *R*^2^ = 0.978) and for *B. subtilis* (0.169, *R*^2^ = 0.720), while that for *B. megaterium* was lower (0.103, *R*^2^ = 0.899). Even the tendency was similar (the adaptation of *B. subtilis* and *B. cereus* was more efficient than *B. megaterium*). These values were a few times smaller when compared with the raw materials of biological origin (Figure 2). 

The use of phosphate rock in the solubilization experiments yielded similar results to those obtained for ashes. The following direction factors of the regression curve, $C_{organic\;acids}^{total}=f\left(dose\right),$ were found: *B. cereus* 0.176 (*R*^2^ = 0.832); *B. subtilis* 0.169 (*R*^2^ = 0.720); *B. megaterium* 0.371 (*R*^2^ = 0.868) (Figure 2). The significantly lower amount of acids produced by *Bacillus* when inorganic sources were used was in line with previous findings [15]. The presence of other biological compounds in poultry and fish bones crucial for metabolism of *Bacillus* resulted in higher amounts of produced acids, as well as different kinds of acids detected in the broth medium. Since ash and phosphate rock are deprived of organic compounds due to the generation process (phosphogenesis in the case of phosphate rock and incineration in the case of ashes) their inorganic nature results in a lower range and concentration of produced organic acids.

## 3. Materials and Methods 

### 3.1. Bacteria and Culture Medium

Phosphate sources were treated with soil bacteria, *Bacillus megaterium* (*B. megaterium*) (PCM 1855), *Bacillus cereus* (*B. cereus*) (PCM 1948), and *Bacillus subtilis* (*B. subtilis*) (PCM 1938), as phosphate-solubilizing microorganisms. The bacteria were obtained from the Polish Collection of Microorganisms located at the Institute of Immunology and Experimental Therapy in Wroclaw (WDCM106). A full description of the procedure, as well as the growth medium composition, was published elsewhere [15]. 

### 3.2. Phosphate Source

Three different concentrations (1, 5, and 30 g/L) of four different sources of phosphorus were used in the conducted solubilization experiments. Three renewable sources (poultry bones, ash from wastewater sludge (from Łyna plant in Olsztyn, Poland), and fish bones) and one non-renewable source (Morocco phosphate rock) were used. The content of phosphorus in the tested P-bearing resources expressed as P_2_O_5_ was as follows: 12.8%—poultry bones; 19.6%—fish bones; 13.4—ash; 34.2%—phosphate rock. P-bearing substrates were ground with a blender and sieved to pass through 1-mm particle-size fractions for solubilization studies.

According to regulation (EC) No 2003/2003 of the European Parliament and of the Council relating to fertilizers (see Sections 3.1.4 Extraction of phosphorus, which is soluble in neutral ammonium citrate, and 3.1.6 Extraction of water-soluble phosphorus), two fractions of phosphorus from phosphate sources used in the experiments were determined: ammonium citrate and water extracts. A full description of the procedure was published elsewhere [15,18].

### 3.3. Experimental and Analytical Methods

**Figure 3:**
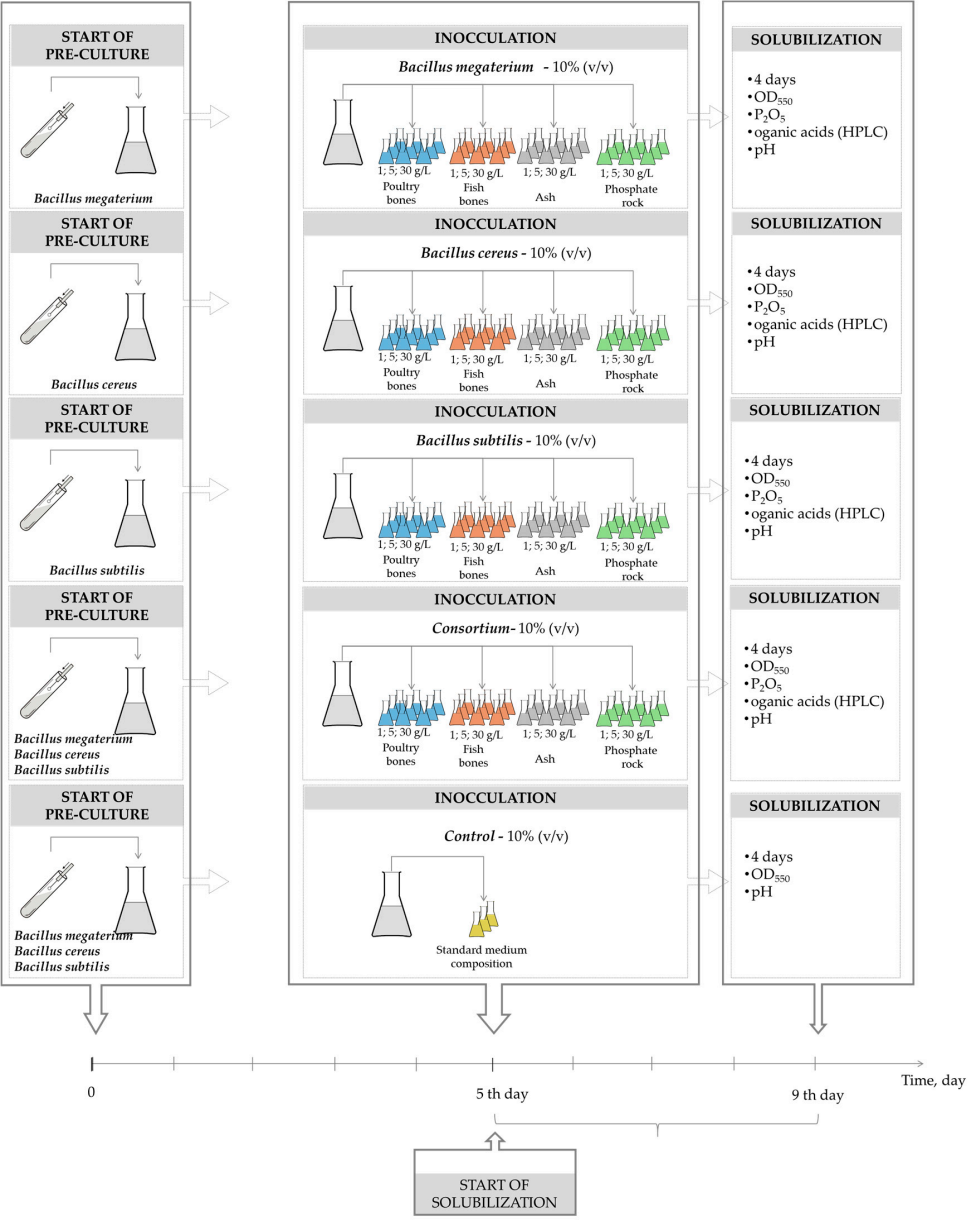
Scheme of performed experiments.

Figure 3 presents the scheme of the conducted experiment. The solubilization experiment was conducted for four days according to a procedure published elsewhere [15]. The measurements were carried out three times. Samples of a microorganism suspension from all culture groups (four groups: poultry cooked bone, fish bones, ash from wastewater sludge, and Morocco phosphate rock) were collected at the same time. The reaction mixture was filtered through filter paper and permeates were used for the estimation of pH, organic acid concentration, and P_2_O_5_ concentration, measured by HPLC and the colorimetric vanadomolybdophosphoric acid method [18]. The biomass concentration of *Bacillus* was measured spectrophotometrically [23]. A full description of the procedure, as well as equations used to evaluate specific growth rate, was published elsewhere [15]. 

The concentrations of organic acids were measured using a high-performance liquid chromatography system (Prominence, Shimadzu, Kioto, Japan) consisting of a Shimadzu LC-20AD pump liquid chromatography SIL-20A autosampler and SPD-M20A diode-array detector. The analytes were separated on a Synergi 4u Hydro-RP 80A C18 column (250 × 4.6 mm, 4 µm) at 35 °C. The separation was carried out by isocratic elution. The mobile phase was prepared by mixing acetonitrile with diluted orthophosphoric acid (pH 2.6, 1% diethylamine) in the appropriate ratio (both solutions were degassed by ultrasonication before use). The flow rate was 1 mL/min. Before measurement, samples were appropriately diluted in deionized water of 18 MΩcm and filtered through a nylon 0.22-µm syringe filter with a diameter of 13 mm (Labe Ltd. Filter-Bio, Nantong, China). Samples were sonicated in an ultrasonic bath. Organic acids in supernatants were determined using a high-performance liquid chromatography (HPLC) method. For quantification of organic acids, the external calibration curve was calculated through the analysis of calibrators at the following concentration levels: 6.25, 12.5, 25, 50, and 100 mg/L. The obtained correlation coefficients of calibration curves were over 0.999. The deionized water was obtained from a Milli Ro & Q water purification system (Merck-Millipore, Billerica, MA, USA). Acetonitrile (ACN, 70%), 99% acetic acid, 99.5% propanoic acid, 99.5%, succinic acid, d-gluconic acid, and sodium salt were produced by Sigma-Aldrich (Steinheim, Germany), while 80% d,l-lactic acid was obtained from Avantor Performance Materials (Gliwice, Poland), and 98% diethylamine and 85% orthophosphoric acid were purchased from Merck (Darmstadt, Germany). 

### 3.4. Calculations

The arithmetic mean values, standard deviations (SD), and *t*-tests, as well as the model parameters of equations describing the experimental data, were determined using nonlinear estimation and multiple regression modules of *Statistica* software ver. 9.0. The correlation was considered statistically significant at α < 0.05. 

The chi-square test (χ^2^ test) was also used, which was calculated from Equation (4), which more accurately described the fit of the model to experimental data compared to the determination coefficient *R*^2^.

(4) $$\chi^{2}=\frac{{\left(\mathrm{experimental}\;\mathrm{value}\;-\;\mathrm{model}\;\mathrm{value}\right)}^{2}}{\mathrm{model}\;\mathrm{value}}$$

## 4. Conclusions

The role of soil biota in the utilization of P-bearing secondary raw materials was described in this paper. The application of phosphate-solubilizing bacteria increases soil fertility due to their ability to convert insoluble P to soluble P through the release of organic acids, chelation, and ion exchange. The microbial solubilization of phosphate rock is a frequently discussed issue in the literature, especially because of its potential application in the mitigation of the phosphorus problem. The action of organic acids is recognized as a major mechanism responsible for phosphorus release from hydroxyapatite structures. Their presence and levels of concentration were described and compared for different types of microbial species. 

Three renewable resources of phosphorus and one non-renewable (phosphate rock) were used as sources of phosphorus in solubilization performed by three different kinds of *Bacillus* species. Solubilizing exudates produced by *Bacillus* were composed of the following five organic acids: gluconic, lactic, acetic, succinic, and propionic. Only *Bacillus megaterium* did not produce propionic acid. A strong correlation between the total concentrations of organic acid and the amounts of released phosphorus P_2_O_2_ was confirmed. Though the highest total organic acid concentration was obtained for a consortium of three *Bacillus* species, the combination of *Bacillus megaterium* with fish bones at 5 g/L was found to be the pair that gave the highest concentration of released phosphorus (483 ± 5 mg/L).

## Figures and Tables

**Table 1:** Monod parameters of growth of *Bacillus* species under different conditions.

Bacteria Strain	Raw Materials	Monod						Haldane					
		Parameter	Unit	Value ± SD	*p*-Value	*R* ^2^	*χ* ^2^	Parameter	Unit	Value ± SD	*p*-Value	*R* ^2^	*χ* ^2^
*B. megaterium*	Bones	*μ_max_*	h^−1^	0.110 ± 0.001 *	0.000	0.999	0.0000169						
		*K_s_*	g/L	0.113 ± 0.014 *	0.016								
	Ash	*μ_max_*	h^−1^	0.129 ± 0.018 *	0.019	0.964	0.00656						
		*K_s_*	g/L	**0.833** ± 0.594	0.296								
	Fish bones	*μ_max_*	h^−1^	0.124 ± 0.007 *	0.003	0.996	0.00103						
		*K_s_*	g/L	1.874 ± 0.426 *	0.048								
	Phosphate rock	*μ_max_*	h^−1^	0.117 ± 0.000 *	0.000	0.996	0.000						
		*K_s_*	g/L	0.100 ± 0.001 *	0.000								
*B. cereus*	Bones	*μ_max_*	h^−1^	0.123 ± 0.001 *	0.000	0.999	0.000007						
		*K_s_*	g/L	0.0465 ± 0.0077 *	0.026								
	Ash	*μ_max_*	h^−1^	**0.135** ± 0.010 *	0.005	0.989	0.00197						
		*K_s_*	g/L	0.182 ± 0.155	0.363								
	Fish bones	*μ_max_*	h^−1^	0.116 ± 0.001 *	0.000	0.999	0.000050						
		*K_s_*	g/L	0.220 ± 0.028 *	0.016								
	Phosphate rock	*μ_max_*	h^−1^	0.119 ± 0.016 *	0.004	0.942	0.0129	*μ_max_*	h^−1^	0.144 ± 0.038	0.064	0.959	0.009
								*K_s_*	g/L	0.438 ± 0.384	0.373		
		*K_s_*	g/L	0.252 ± 0.207	0.310			*K_I_*	g/L	85.9 ± 119	0.546		
*B. subtilis*	Bones	*μ_max_*	h^−1^	0.126 ± 0.003 *	0.000	0.998	0.000207						
		*K_s_*	g/L	0.0947 ± 0.0456	0.173								
	Ash	*μ_max_*	h^−1^	**0.127** ± 0.007 *	0.003	0.993	0.00112						
		*K_s_*	g/L	0.112 ± 0.109	0.412								
	Fish bones	*μ_max_*	h^−1^	0.120 ± 0.011 *	0.008	0.984	0.00253						
		*K_s_*	g/L	0.525 ± 0.285	0.207								
	Phosphate rock	*μ_max_*	h^−1^	0.104 ± 0.018 *	0.009	0.998	0.0181	*μ_max_*	h^−1^	0.129 ± 0.045	0.101	0.935	0.013
		*K_s_*	g/L	0.233 ± 0.253	0.425			*K_s_*	g/L	0.442 ± 0.496	0.467		
								*K_I_*	g/L	69.3 ± 109	0.590		

Type of raw material: poultry bones, ash, fish bones, or phosphate rock; dose of phosphorus raw material: 1, 5, or 30 g/L. *: statistically significant parameters; numbers in bold: the highest values within the group.

**Table 2:** Parameters of model $C_{P_{2}O_{5}}=f\left(t\right)=\frac{C_{P_{2}O_{5}}^{max}}{1+b\cdot exp^{-k\cdot t}}$ describing the kinetics of changes in P_2_O_5_ concentration during solubilization.

Bacteria Strain	Dose		Bones				Fish Bones				Ash				Phosphate Rock			
		Parameter	Value ± SD	*p*-Value	*R* ^2^	χ^2^	Value ± SD	*p*-Value	*R* ^2^	χ^2^	Value ± SD	*p*-Value	*R* ^2^	χ^2^	Value ± SD	*p*-Value	*R* ^2^	χ^2^
*B. megaterium*	1 g/L	$C_{P_{2}O_{5}}^{max}$ (mg/L)	133 ± 2 *	0.000	0.999	0.117	213 ± 6	0.001	0.987	0.987	47.5 ± 1.7 *	0.001	0.995	0.258	37.0 ± 1.5 *	0.002	0.990	0.0819
		*b* (day)	3.14 ± 0.18 *	0.003			0.999 ± 0.199	0.037			1.94 ± 0.31 *	0.024			0.628 ± 0.082 *	0.016		
		*k* (1/day)	0.0399 ± 0.0020 *	0.002			0.0896 ± 0.0252	0.071			0.0391 ± 0.0065 *	0.026			0.0216 ± 0.0056	0.061		
	5 g/L	$C_{P_{2}O_{5}}^{max}$ (mg/L)	270 ± 11 *	0.002	0.998	2.54	**483 ± 5**	**0.000**	0.998	0.128	85.4 ± 1.7 *	0.000	0.999	0.0676	33.4 ± 1.4 *	0.002	0.994	0.102
		*b* (day)	7.25 ± 0.99 *	0.018			0.565 ± 0.028	0.002			3.26 ± 0.14 *	0.002			1.31 ± 0.16 *	0.014		
		*k* (1/day)	0.0287 ± 0.0029 *	0.009			0.0304 ± 0.0026	0.007			0.0238 ± 0.0012 *	0.003			0.0257 ± 0.0047 *	0.032		
	30 g/L	$C_{P_{2}O_{5}}^{max}$ (mg/L)	279 ± 6 *	0.000	0.999	0.591	433 ± 12	0.001	0.985	0.775	43.6 ± 1.2 *	0.001	0.999	0.0376	29.5 ± 3.2 *	0.011	0.982	0.338
		*b* (day)	73.5 ± 6.4 *	0.007			0.408 ± 0.061	0.022			1.56 ± 0.08 *	0.003			1.61 ± 0.35 *	0.043		
		*k* (1/day)	0.0355 ± 0.0012 *	0.001			0.0284 ± 0.0082	0.074			0.0200 ± 0.0018 *	0.008			0.0206 ± 0.0073	0.106		
*B. cereus*	1 g/L	$C_{P_{2}O_{5}}^{max}$ (mg/L)	67.8 ± 2.86 *	0.002	0.970	0.345	221 ± 5	0.001	0.996	0.241	33.5 ± 0.7 *	0.000	0.998	0.0388	35.1 ± 1.8 *	0.003	0.993	0.999
		*b* (day)	0.523 ± 0.122	0.051			0.834 ± 0.073	0.008			1.95 ± 0.18 *	0.008			1.09 ± 0.13 *	0.014		
		*k* (1/day)	0.0362 ± 0.0144	0.128			0.0279 ± 0.0040	0.020			0.0410 ± 0.0038 *	0.009			0.0203 ± 0.0044 *	0.043		
	5 g/L	$C_{P_{2}O_{5}}^{max}$ (mg/L)	76.8 ± 4.9 *	0.004	0.975	1.29	393 ± 16	0.002	0.987	3.23	53.1 ± 3.3 *	0.004	0.993	0.524	33.9 ± 2.8 *	0.007	0.994	0.131
		*b* (day)	1.33 ± 0.392	0.077			1.93 ± 0.55	0.073			3.06 ± 0.58 *	0.034			1.57 ± 0.20 *	0.016		
		*k* (1/day)	0.0404 ± 0.0141	0.104			0.0890 ± 0.0223	0.057			0.0285 ± 0.0056 *	0.037			0.0163 ± 0.0037 *	0.047		
	30 g/L	$C_{P_{2}O_{5}}^{max}$ (mg/L)	42.6 ± 4.25 *	0.010	0.976	2.21	405 ± 5	0.000	0.998	0.347	42.7 ± 2.8 *	0.004	0.991	0.210	31.5 ± 6.4 *	0.038	0.981	0.330
		*b* (day)	3.33 ± 1.43	0.145			1.2364 ± 0.105419	0.007			1.41 ± 0.20 *	0.020			1.63 ± 0.46	0.071		
		*k* (1/day)	0.0363 ± 0.0138	0.119			0.0855 ± 0.0087	0.010			0.0214 ± 0.0052	0.054			0.0145 ± 0.0067	0.164		
*B. subtilis*	1 g/L	$C_{P_{2}O_{5}}^{max}$ (mg/L)	79.4 ± 13.3 *	0.027	0.993	0.445	242 ± 26	0.011	0.962	4.09	42.1 ± 1.2 *	0.001	0.996	0.0860	33.1 ± 2.8 *	0.007	0.977	0.293
		*b* (day)	2.12 ± 0.44 *	0.041			0.981 ± 0.285	0.075			1.06 ± 0.10 *	0.009			1.03 ± 0.23 *	0.047		
		*k* (1/day)	0.0136 ± 0.0039	0.076			0.0221 ± 0.0115	0.194			0.0273 ± 0.0041 *	0.022			0.0224 ± 0.0089	0.127		
	5 g/L	$C_{P_{2}O_{5}}^{max}$ (mg/L)	56.6 ± 3.6 *	0.004	0.944	0.791	427 ± 10	0.001	0.993	0.886	36.6 ± 1.5 *	0.002	0.988	0.274	32.0 ± 5.3 *	0.026	0.964	0.382
		*b* (day)	0.668 ± 0.235	0.105			0.842 ± 0.112	0.017			1.34 ± 0.28 *	0.040			0.939 ± 0.298	0.088		
		*k* (1/day)	0.0429 ± 0.0229	0.202			0.0522 ± 0.0095	0.031			0.0453 ± 0.0106	0.051			0.0155 ± 0.0096	0.247		
	30 g/L	$C_{P_{2}O_{5}}^{max}$ (mg/L)	70.2 ± 34.5	0.179	0.958	1.504	428 ± 14	0.001	0.984	2.27	51.0 ± 2.9 *	0.003	0.985	0.665	41.2 ± 6.8 *	0.026	0.994	0.0622
		*b* (day)	1.79 ± 1.17	0.266			0.900 ± 0.195	0.044			1.65 ± 0.41	0.056			1.16 ± 0.32	0.068		
		*k* (1/day)	0.0118 ± 0.0098	0.353			0.0894 ± 0.0294	0.093			0.0384 ± 0.0106	0.069			0.00983 ± 0.00341	0.102		
Consortium	30 g/L	$C_{P_{2}O_{5}}^{max}$ (mg/L)	106 ± 4 *	0.002	0.992	0.719	414 ± 9	0.001	0.994	0.934	27.3 ± 1.3 *	0.002	0.978	0.106	36.7 ± 6.9 *	0.034	0.989	0.0836
		*b* (day)	2.24 ± 0.469 *	0.041			1.08 ± 0.15	0.018			0.549 ± 0.104 *	0.034			0.918 ± 0.323	0.105		
		*k* (1/day)	0.0430 ± 0.0085 *	0.037			0.0680 ± 0.0114	0.027			0.0248 ± 0.0090	0.111			0.00973 ± 0.00470	0.174		

*: statistically significant parameters, numbers in bold: the highest value.

**Table 3:** The levels of detected organic acid concentrations (mmol/L) in the culture broth of *Bacillus* species under different conditions.

Bacteria Strain	Raw Material	Dose g/L	Gluconic Acid			2-Hydroxypropanoic Acid (Lactic Acid)			Acetic Acid			Butanedoic Acid (Succinic Acid)			Propionic Acid		
			Con. ± SD	f(dose) = Con.		Con. ± SD	f(dose) = Con.		Con. ± SD	f(dose) = Con.		Con. ± SD	f(dose) = Con.		Con. ± SD	f(dose) = Con.	
				*R*	*p*		*R*	*p*		*R*	*p*		*R*	*p*		*R*	*p*
*B. megaterium*	PB	1	3.15 ± 0.03	0.940	0.222	1.32 ± 0.03 *	0.998	0.043	2.83 ± 0.06	−0.463	0.694	<LOQ	-	-	<LOQ	-	-
		5	7.13 ± 0.19			2.12 ± 0.18 *			2.97 ± 0.02			1.63 ± 0.03			<LOQ		
		30	11.8 ± 0.1			5.20 ± 0.07 *			2.82 ± 0.02			8.63 ± 0.01			<LOQ		
	A	1	0.542 ± 0.049	−0.725	0.483	0.849 ± 0.034	0.981	0.124	1.38 ± 0.05	0.636	0.562	0.660 ± 0.023	-	-	<LOQ	-	-
		5	0.135 ± 0.004			1.78 ± 0.03			3.01 ± 0.06			<LOQ			<LOQ		
		30	0.051 ± 0.006			3.71 ± 0.08			3.09 ± 0.17			<LOQ			<LOQ		
	FB	1	5.14 ± 0.20	-	-	4.05 ± 0.09	0.992	0.080	1.22 ± 0.06	0.469	0.689	1.19 ± 0.01	0.994	0.071	<LOQ	-	-
		5	9.71 ± 0.09			10.1 ± 0.2			4.28 ± 0.07			1.39 ± 0.02			<LOQ		
		30	<LOQ			27.5 ± 0.1			3.75 ± 0.02			11.8 ± 0.02			<LOQ		
	PR	1	0.375 ± 0.046	0.853	0.350	0.992 ± 0.054	0.971	0.153	3.06 ± 0.04	0.709	0.498	<LOQ	-	-	<LOQ	-	-
		5	0.579 ± 0.502			4.34 ± 0.65			5.32 ± 0.62			0.169 ± 0.014			<LOQ		
		30	0.697 ± 0.041			10.2 ± 0.1			5.72 ± 0.11			0.331 ± 0.018			<LOQ		
*B. cereus*	PB	1	0.608 ± 0.037	-	-	2.69 ± 0.40 *	0.999	0.031	<LOQ	-	-	<LOQ	-	-	<LOQ	-	-
		5	<LOQ			8.91 ± 0.48 *			<LOQ			<LOQ			0.439 ± 0.007		
		30	<LOQ			36.0 ± 0.4 *			<LOQ			<LOQ			1.89 ± 0.015		
	A	1	0.589 ± 0.026	0.522	0.650	2.26 ± 0.18	0.995	0.062	<LOQ	-	-	<LOQ	-	-	<LOQ	-	-
		5	0.393 ± 0.008			2.37 ± 0.05			<LOQ			<LOQ			<LOQ		
		30	0.628 ± 0.008			5.26 ± 0.10			1.12 ± 0.05			<LOQ			<LOQ		
	FB	1	<LOQ	-	-	4.38 ± 0.06	0.980	0.128	15.2 ± 0.3	−0.273	0.824	<LOQ	-	-	<LOQ	-	-
		5	1.24 ± 0.06			14.6 ± 0.1			2.57 ± 0.06			0.390 ± 0.108			0.334 ± 0.008		
		30	<LOQ			35.5 ± 0.3			7.25 ± 0.04			0.709 ± 0.022			0.690 ± 0.044		
	PR	1	0.329 ± 0.015	−0.669	0.533	3.10 ± 0.19	0.959	0.184	<LOQ	-	-	<LOQ	-	-	<LOQ	-	-
		5	0.318 ± 0.001			5.28 ± 0.10			0.985 ± 0.076			<LOQ			<LOQ		
		30	0.316 ± 0.005			8.48 ± 0.09			0.685 ± 0.024			<LOQ			<LOQ		
*B. subtilis*	PB	1	0.391 ± 0.021	-	-	3.84 ± 0.07 *	0.999	0.024	<LOQ	-	-	<LOQ	-	-	<LOQ	-	-
		5	<LOQ			7.73 ± 0.06 *			4.39 ± 0.19			<LOQ			2.67 ± 0.05		
		30	<LOQ			25.9 ± 0.9 *			13.8 ± 0.8			0.499 ± 0.037			0.565 ± 0.019		
	A	1	0.422 ± 0.001 *	−0.997	0.045	1.93 ± 0.08	−0.651	0.549	<LOQ	-	-	<LOQ	-	-	<LOQ	-	-
		5	0.413 ± 0.018 *			3.46 ± 0.01			2.41 ± 0.08			<LOQ			<LOQ		
		30	0.376 ± 0.007 *			1.22 ± 0.02			5.96 ± 0.07			0.302 ± 0.007			0.652 ± 0.055		
	FB	1	<LOQ	-	-	3.22 ± 0.04	0.988	0.098	2.17 ± 0.08	0.749	0.461	0.097 ± 0.011	0.565	0.618	<LOQ	-	-
		5	0.646 ± 0.014			12.8 ± 0.1			5.30 ± 0.06			0.409 ± 0.020			<LOQ		
		30	<LOQ			36.7 ± 0.4			6.11 ± 0.21			0.391 ± 0.056			<LOQ		
	PR	1	0.426 ± 0.024	−0.655	0.545	4.31 ± 0.05	−0.071	0.954	<LOQ	-	-	<LOQ	-	-	<LOQ	-	-
		5	0.228 ± 0.001			3.39 ± 0.16			7.79 ± 0.28			0.097 ± 0.004			<LOQ		
		30	0.212 ± 0.003			3.90 ± 0.13			3.92 ± 0.08			0.234 ± 0.017			<LOQ		
Consortium	PB	30	<LOQ	-	-	23.1 ± 0.2	-	-	12.9 ± 0.3	-	-	0.356 ± 0.060	-	-	0.751 ± 0.010	-	-
	A	30	0.370 ± 0.019			4.63 ± 0.17			4.58 ± 0.26			<LOQ			<LOQ		
	FB	30	<LOQ			37.7 ± 0.1			10.1 ± 0.5			0.668 ± 0.003			0.485 ± 0.015		
	FR	30	0.315 ± 0.027			7.99 ± 0.15			5.17 ± 0.23			0.255 ± 0.005			<LOQ		

Type of raw material: poultry bones (PB), ash (A), fish bones (FB), or phosphate rock (FR); dose of phosphorus raw material: 1, 5, or 30 g/L; correlation parameters (*R*) and *p*-values represent the relationship between the dose and the concentrations of synthesized acid. *: statistically significant parameters. LOQ: limit of quantification, -: not applicable.

**Table 4:** The relationship between the total organic acid concentration and P_2_O_5_ concentration in the solution.

Bacteria	Dose (g/L)	$C_{P_{2}O_{5}}=f\left(C_{organic\;acids}^{total}\right)$	*R*	*p*-Value	*R* ^2^
*B. cereus*	1	$C_{P_{2}O_{5}}$ = −11.2 + 10.7 · C*_acids_*	0.985	0.014	0.971
	5	$C_{P_{2}O_{5}}$ = −76.3 + 22.7 · C*_acids_*	0.936	0.064	0.876
	30	$C_{P_{2}O_{5}}$ = −31.6 + 6.6 · C*_acids_*	0.688	0.312	0.473
*B. megaterium*	1	$C_{P_{2}O_{5}}$ = −40.3 + 22.1 · C*_acids_*	0.982	0.0177	0.965
	5	$C_{P_{2}O_{5}}$ = −77.1 + 21.6 · C*_acids_*	0.922	0.0777	0.851
	30	$C_{P_{2}O_{5}}$ = −88.2 + 11.9 · C*_acids_*	0.953	0.0468	0.909
*B. subtilis*	1	$C_{P_{2}O_{5}}$ = −98.6 + 47.1 · C*_acids_*	0.647	0.353	0.419
	5	$C_{P_{2}O_{5}}$ = −224 + 28 · C*_acids_*	0.787	0.213	0.619
	30	$C_{P_{2}O_{5}}$ = −13.4 + 6.4 · C*_acids_*	0.663	0.337	0.439

## Supplementary Materials

The following are available online.
