# Supplementary material for: Phosphorus Solubilization by Bacillus Species
Source: Molecules. 2018 Nov 6;23(11):2897. doi: 10.3390/molecules23112897 (PMC6278551; doi:10.3390/molecules23112897)
Supplement: Supplementary file 1 [file molecules-23-02897-s001.pdf]

molecules

Table S1. The equation $\mu = f(t)$ describing the specific growth rate of *Bacillus* species under different conditions. Type of raw material: poultry bones, ash, fish bones and phosphate rock; dose of phosphorus raw material: 1, 5 and 30 g/L; ↓↑ - decrease/increase of μ with the increase of dose applied.

Bacteria	Raw materials	Concentration, g/L	μ, 1/h		$\mu = f(t)$	R^2	ΔpH
B. megaterium	Bones	1	0.099		$\mu = 0.099t + 0.727$	0.781	-1.47
		5	0.107	↑	$\mu = 0.107t + 0.58$	0.866	-1.79
		30	0.1105		$\mu = 0.1105t + 0.517$	0.898	-1.24
	Ash	1	0.079		$\mu = 0.079t + 0.147$	0.982	-1.77
		5	0.0896	↑	$\mu = 0.0896t - 0.103$	0.993	-2.07
		30	**0.139**		$\mu = 0.139t + 1.026$	0.779	-2.15
	Fish bones	1	0.0385		$\mu = 0.0385t + 0.695$	0.861	0.15
		5	0.097	↑	$\mu = 0.097t + 0.741$	0.765	-0.06
		30	0.114		$\mu = 0.114t + 0.693$	0.838	0.18
	Phosphate rock	1	0.106		$\mu = 0.106t + 0.692$	0.818	-1.93
		5	0.114	↑	$\mu = 0.114t + 0.635$	0.862	-2.45
		30	0.116		$\mu = 0.116t + 0.657$	0.858	-1.34
B. cereus	Bones	1	0.117		$\mu = 0.117t + 0.768$	0.818	-2.03
		5	0.121	↑	$\mu = 0.121t + 0.698$	0.852	-1.13
		30	0.123		$\mu = 0.123t + 0.582$	0.896	-0.29
	Ash	1	0.117		$\mu = 0.117t + 0.8173$	0.796	-1.44
		5	0.117	↑	$\mu = 0.117t + 0.745$	0.827	-2.14
		30	**0.143**		$\mu = 0.143t + 0.932$	0.870	-1.01
	Fish bones	1	0.095		$\mu = 0.095t + 0.619$	0.820	-0.2
		5	0.109	↑	$\mu = 0.109t + 0.838$	0.765	1.21
		30	0.116		$\mu = 0.116t + 0.491$	0.915	1.53
	Phosphate rock	1	0.121		$\mu = 0.121t + 0.872$	0.788	-1.92
		5	0.116	↓	$\mu = 0.116t + 0.864$	0.780	-1.08
		30	0.107		$\mu = 0.107t + 0.642$	0.842	-1.08
B. subtilis	Bones	1	0.116		$\mu = 0.116t + 0.911$	0.758	-2.09
		5	0.120	↑	$\mu = 0.120t + 0.953$	0.753	-1.38
		30	0.129		$\mu = 0.129t + 1.008$	0.759	-0.55
	Ash	1	0.116		$\mu = 0.116t + 0.626$	0.868	-1.69
		5	0.115	↑	$\mu = 0.115t + 0.76$	0.813	-1.28
		30	**0.134**		$\mu = 0.134t + 0.581$	0.910	-1.61
	Fish bones	1	0.0833		$\mu = 0.0833t + 0.690$	0.737	2.02
		5	0.096	↑	$\mu = 0.096t + 0.927$	0.673	0.92
		30	0.127		$\mu = 0.127t - 0.107$	0.996	0.36
	Phosphate rock	1	0.114		$\mu = 0.114t + 0.876$	0.766	-2.21
		5	0.101	↓	$\mu = 0.101t + 0.926$	0.697	-1.06
		30	0.092		$\mu = 0.092t + 0.770$	0.731	-0.14
**Consortium:**	Bones	30	0.123		$\mu = 0.1231t + 0.856$	0.799	-0.39
B. megaterium	Ash	30	0.116		$\mu = 0.116t + 0.894$	0.763	-1.82
B. cereus	Fish bones	30	0.132		$\mu = 0.132t + 0.278$	0.978	1.28
B. subtilis	Phosphate rock	30	0.086		$\mu = 0.086t + 0.879$	0.648	0.45
**Consortium**	Standard medium		0.130		$\mu = 0.130t + 0.739$	0.857	2.66

Table S2 The correlation matrix between the pH and P_2O_5 and SF; N=41, * – statistically significant.

Time, h		pH				
		0	24	48	96	168
P_2O_5	0	*-0.824** *p=0.000*	*-0.311** *p=0.047*	-0.199 p=0.213	0.054 p=0.738	0.198 p=0.215
	24	*-0.797** *p=0.000*	*-0.389** *p=0.012*	-0.268 p=0.090	-0.017 p=0.918	0.109 p=0.495
	48	*-0.751** *p=0.000*	*-0.405** *p=0.009*	*-0.308** *p=0.050*	-0.060 p=0.709	0.060 p=0.708
	96	*-0.708** *p=0.000*	*-0.424** *p=0.006*	*-0.355** *p=0.023*	-0.137 p=0.392	-0.053 p=0.742
	168	*-0.595** *p=0.000*	*-0.410** *p=0.008*	*-0.382** *p=0.014*	-0.231 p=0.145	-0.202 p=0.205
SF	0	*-0.656** *p=0.000*	*-0.477** *p=0.002*	*-0.438** *p=0.002*	*-0.322** *p=0.040*	-0.138 p=0.389
	24	*-0.694** *p=0.000*	*-0.493** *p=0.001*	*-0.449** *p=0.003*	-0.287 p=0.069	-0.126 p=0.432
	48	*-0.677** *p=0.000*	*-0.497** *p=0.001*	*-0.479** *p=0.002*	*-0.313** *p=0.046*	-0.163 p=0.307
	96	*-0.634** *p=0.000*	*-0.481** *p=0.001*	*-0.499** *p=0.001*	*-0.371** *p=0.017*	-0.277 p=0.080
	168	*-0.581** *p=0.000*	*-0.479** *p=0.001*	*-0.519** *p=0.001*	*-0.415** *p=0.007*	*-0.342** *p=0.028*
